# Surgical resection of arteriovenous malformation of the pancreatic head with acute pancreatitis: a case report

**DOI:** 10.1093/jscr/rjac427

**Published:** 2022-09-28

**Authors:** Hiroyuki Hakoda, Yoshikuni Kawaguchi, Yoichi Miyata, Junichi Togashi, Motoki Nagai, Yoshio Suzuki, Yukihiro Nomura

**Affiliations:** Department of Surgery, Asahi General Hospital, Chiba, Japan; Hepato-Biliary-Pancreatic Surgery Division, Department of Surgery, Graduate School of Medicine, The University of Tokyo, Tokyo, Japan; Department of Surgery, Asahi General Hospital, Chiba, Japan; Department of Surgery, Asahi General Hospital, Chiba, Japan; Department of Surgery, Asahi General Hospital, Chiba, Japan; Department of Pathology, Asahi General Hospital, Chiba, Japan; Department of Surgery, Asahi General Hospital, Chiba, Japan

**Keywords:** pancreaticoduodenectomy, arteriovenous fistula, pancreas, surgery

## Abstract

Arteriovenous malformations (AVMs) are uncommon in the gastrointestinal tract, particularly in the pancreas. AVMs cause complications, including gastrointestinal bleeding, portal hypertension and pancreatitis. Therefore, a treatment strategy is not yet established. Surgical treatment or transcatheter arterial embolization is performed in patients with AVM, considering their conditions. A 54-year-old man presented with acute abdominal pain was diagnosed with acute pancreatitis due to AVM of the pancreatic head using dynamic computed tomography. Endoscopic ultrasonography further revealed meandering blood vessels in the pancreatic head. The patient underwent subtotal stomach-preserving pancreaticoduodenectomy. Histological examination revealed AVM of the pancreatic head with chronic pancreatitis. The patient had a good postoperative clinical course and was discharged on postoperative day 22. He remained asymptomatic. Pancreaticoduodenectomy can be considered an effective treatment method for selected cases of symptomatic AVM of the pancreatic head.

## INTRODUCTION

Although arteriovenous malformations (AVMs) of the gastrointestinal tract are commonly found in the cecum and right colon, AVMs of the pancreas are uncommon [1–4]. AVMs cause various complications, including gastrointestinal bleeding, pancreatitis and portal hypertension. However, previous studies report no predominant location in the pancreas [5, 6]. The number of reports of pancreatic AVMs is increasing because of the development of diagnostic imaging modalities [7, 8]. However, the treatment strategy for pancreatic AVM remains unknown [2]. Herein, we report a case of a 54-year-old male who had an AVM at the pancreatic head with acute pancreatitis and underwent surgery.

## CASE REPORT

**Figure 1:**
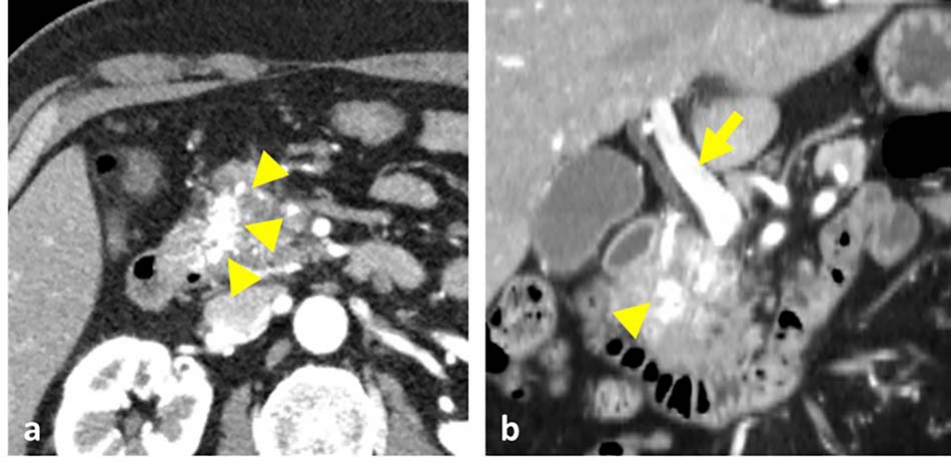
Contrast-enhanced CT images. (**a**) Vessels in the pancreatic head were strongly enhanced during the arterial phase (arrowheads). (**b**) Both the vessels in the pancreas head (arrowhead) and the portal vein (arrow) were enhanced at the arterial phase.

**Figure 2:**
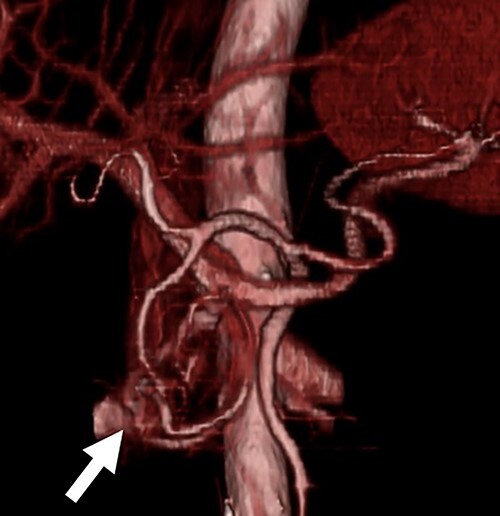
Three-dimensional CT image. The three-dimensional CT imaging showed the communication between arteries and veins in the pancreatic head (arrow).

**Figure 3:**
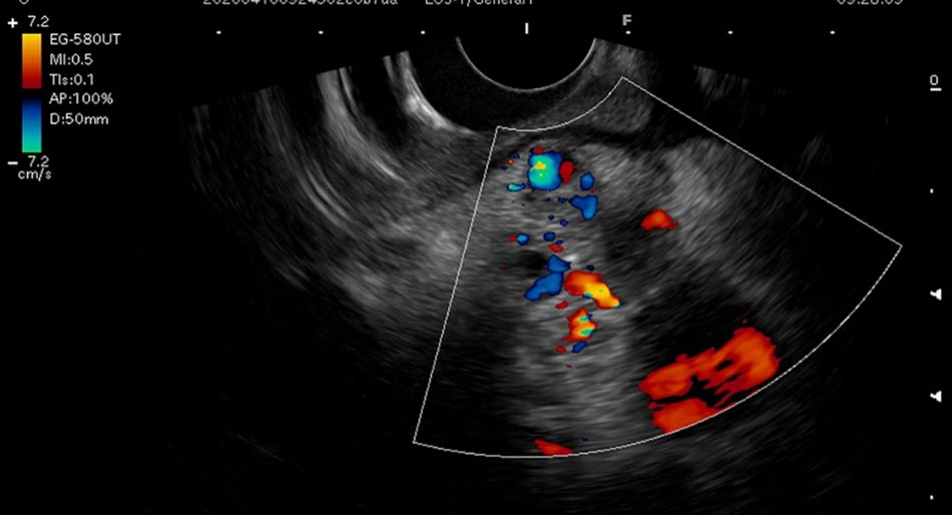
Endoscopic ultrasonography image. Endoscopic ultrasonography showed tortuous vessels and low echoic lesions in the pancreas head, and no findings suspicious of malignancy.

A 54-year-old man was admitted to our hospital presenting with acute upper abdominal pain. He had no family history of pancreatitis, hereditary disease or abdominal trauma; however, he had a history of habitual alcohol consumption. Blood tests showed an elevated white blood cell count (16 900/μl), serum amylase (260 U/l) and lipase (337 U/l). Contrast-enhanced computed tomography (CT) revealed meandering vessels in the pancreatic head. These vessels were enhanced in both the arterial and portal phases, while the portal vein was enhanced in the arterial phase. Swelling of the pancreatic head suggested the development of pancreatitis (Fig. 1). Three-dimensional CT revealed blood vessel communications between the arteries and veins in the pancreas (Fig. 2). Endoscopic ultrasonography (EUS) also revealed meandering vessels in the pancreatic head surrounded by low echoic lesions with diameters of 8 mm (Fig. 3). Magnetic resonance imaging (MRI) revealed flow voids and a cyst measuring 8 mm in diameter at the pancreatic head. Neither stenosis nor dilation of the main pancreatic duct was observed.

**Figure 4:**
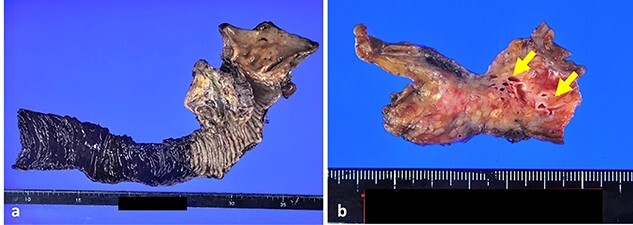
Gross appearance of specimens. (**a**) Gross appearance of pancreaticoduodenectomy specimen. (**b**) Slice of the pancreatic head showed dilated meandering blood vessels (arrows).

**Figure 5:**
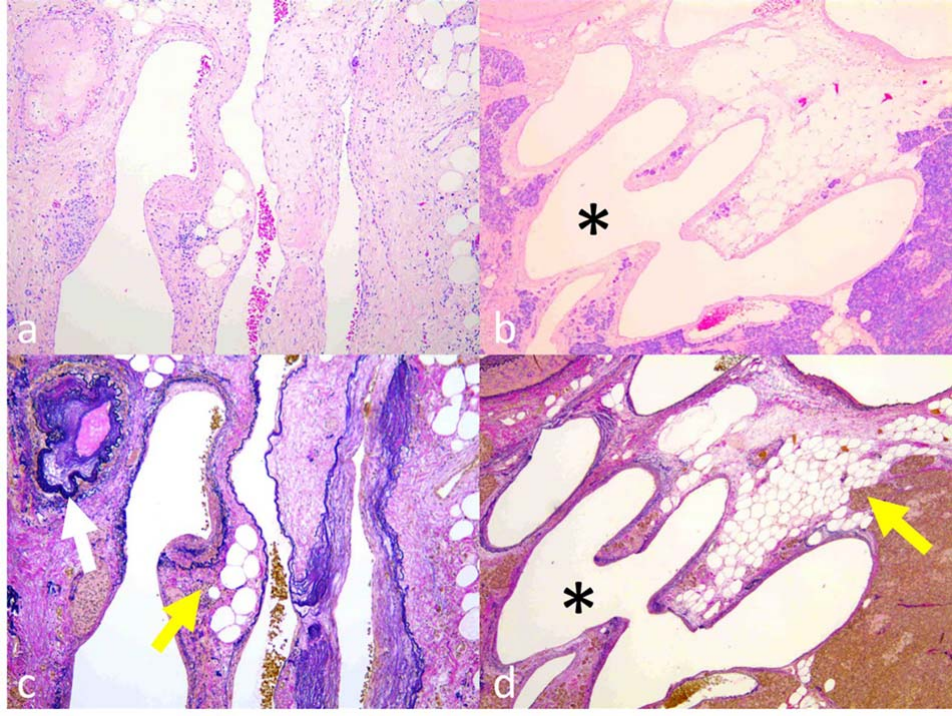
Histopathological findings. (**a**, **b**) Dilated and tortuous veins and arteries. The transition from an artery to a vein was detected (b, asterisk). (**c**, **d**) Development of elastic fibers with thickening wall of arteries (c, colorless arrow) and dilated veins with steatosis of the pancreas parenchyma (c and d, colored arrow). (a, b) Hematoxylin–eosin staining; (c, d) Elastica van Gieson staining).

Based on these imaging findings, the patient was diagnosed with AVM in the pancreatic head and subsequently underwent subtotal stomach-preserving pancreaticoduodenectomy. Intraoperative ultrasonography revealed tortuous vessels in the head of the pancreas. Severe inflammation around the pancreatic head and adhesion between the pancreas and portal vein increased the complexity of the surgical procedure and hindered inflow control of the pancreas. To control bleeding, we first ligated and divided the arterial inflow into the pancreatic head. The operative time was 712 min, and the estimated blood loss was 1260 ml. Grossly, the specimens had dilated and tortuous blood vessels in the pancreatic head, development of elastic fibers with thickened arterial walls and dilated veins, and pancreatic parenchyma steatosis (Fig. 4). The transition from arteries to veins was not detected macroscopically. Microscopic examination similarly revealed the presence of dilated vessels in the pancreatic head, the majority of which were veins containing few smooth muscles and thick and ruptured elastic fibers (Fig. 5). Based on these histopathological findings, the postoperative diagnosis was AVM of the pancreatic head and pancreatitis.

The postoperative course was uneventful, except for a postoperative pancreatic fistula of grade B according to the classification of the International Study Group of Pancreatic Fistula [9]. The patient was discharged on postoperative day 22. He remained asymptomatic and showed no postoperative recurrence.

## DISCUSSION

AVMs are reportedly found in the cecum and right colon [3]. AVMs of the pancreas are rare, accounting for only 0.9% of all AVM cases [1, 2, 10–12]. Previous reports showed that 48.3–62.3% of pancreatic AVM were found at the pancreatic head, followed by the pancreatic body and tail, and the entire pancreas [1, 5, 6]. Further, studies showed that the main cause of pancreatic AVM was congenital in origin, associated with Osler–Weber–Rendu disease, accounting for approximately 90% of causes of pancreatic AVM [5, 13]. Other causes include trauma, pancreatitis and tumors [2, 4]. Most AVM cases are asymptomatic; however, with pancreatic AVM, patients may present with upper abdominal pain, gastrointestinal bleeding, portal hypertension and pancreatitis [1, 4, 5, 7, 11, 14]. Portal hypertension develops when the AVM of the pancreas grows progressively in size [2]. When associated with pancreatitis, this can cause further gastrointestinal ulcers, varices and bleeding [10]. Our case was unique because the pancreatic AVM, presenting as acute upper abdominal pain, was caused by acute chronic pancreatitis without a family history of Osler–Weber–Rendu disease and refractory duodenal ulcer. Further, portal hypertension was not suspected according to the imaging modalities before and after the operation.

Angiography is the definitive diagnostic modality for AVM, showing the direct communication between the arteries and veins; however, its drawback is its invasiveness. Hence, other diagnostic modalities [7, 8], including CT, MRI and EUS, are used to safely and accurately diagnose AVM of the pancreas [1–3, 13]. Particularly, three-dimensional imaging reconstructed using CT is useful for the diagnosis of AVM. The patient in our case was accurately diagnosed with an AVM of the pancreatic head using contrast-enhanced CT and EUS without angiography.

Surgical resection, transarterial embolization (TAE) and irradiation are reported for the treatment of pancreatic AVM [5, 10, 14]. TAE is a less invasive procedure than surgery [15]; however, it is associated with a high risk of AVM recurrence and embolization of other portal branches because of the migration of embolic agents [5]. Moreover, because the AVM is fed by multiple arteries, it is difficult to embolize all feeding arteries [6, 13, 14]. Studies have recommended early surgical resection of the pancreatic AVM to prevent portal hypertension, an irreversible change even after resection and its sequelae [3, 6, 13].

In our case, TAE may have been a treatment option; however, multiple feeding arteries were a hurdle for complete embolization. Surgery of the pancreatic AVM was a curative treatment and improved his quality of life, with no recurrence of abdominal pain post-surgery.

## CONCLUSIONS

Pancreaticoduodenectomy is an effective and curative treatment for patients with AVM of the pancreatic head. Surgical resection should be considered before portal hypertension develops because portal hypertension is irreversible even after treatment.

## Data Availability

The data that support the findings of this study are available on reasonable request from the corresponding author.
